# Clinical Impact of Antibiotics for the Treatment of *Pseudomonas aeruginosa* Biofilm Infections

**DOI:** 10.3389/fmicb.2019.02894

**Published:** 2020-01-09

**Authors:** Elodie Olivares, Stéphanie Badel-Berchoux, Christian Provot, Gilles Prévost, Thierry Bernardi, François Jehl

**Affiliations:** ^1^University of Strasbourg, CHRU Strasbourg, Fédération de Médecine Translationnelle de Strasbourg, EA7290, Institut de Bactériologie, Strasbourg, France; ^2^BioFilm Pharma SAS, Saint-Beauzire, France; ^3^BioFilm Control SAS, Saint-Beauzire, France

**Keywords:** biofilms, antibiotic tolerance, biofilm-related infections, *Pseudomonas aeruginosa*, clinical laboratory technique, MBEC assay, antibiofilmogram

## Abstract

Bacterial biofilms are highly recalcitrant to antibiotic therapies due to multiple tolerance mechanisms. The involvement of *Pseudomonas aeruginosa* in a wide range of biofilm-related infections often leads to treatment failures. Indeed, few current antimicrobial molecules are still effective on tolerant sessile cells. In contrast, studies increasingly showed that conventional antibiotics can, at low concentrations, induce a phenotype change in bacteria and consequently, the biofilm formation. Understanding the clinical effects of antimicrobials on biofilm establishment is essential to avoid the use of inappropriate treatments in the case of biofilm infections. This article reviews the current knowledge about bacterial growth within a biofilm and the preventive or inducer impact of standard antimicrobials on its formation by *P. aeruginosa*. The effect of antibiotics used to treat biofilms of other bacterial species, as *Staphylococcus aureus* or *Escherichia coli*, was also briefly mentioned. Finally, it describes two *in vitro* devices which could potentially be used as antibiotic susceptibility testing for adherent bacteria.

## Introduction

Bacterial biofilm was defined for the first time in 1978 as a structured community of microorganisms adhering to a surface and producing an extracellular matrix of polysaccharides (Costerton et al., 1978). It represents a particular behavior of bacteria triggered by the proximity of a surface and involving complex signaling networks, including quorum sensing (QS). Its discovery was attributed to the microscope inventor, Antoni Van Leeuwenhoek who observed bacteria clusters on dental plaque in 1684. He wrote in a report for the Royal Society of London: “The number of these animalcules in the scurf of a man’s teeth are so many that I believe they exceed the number of men in a kingdom” (Biofilms: The Hypertextbook, 2011).

Nowadays, it is well-recognized that biofilms play an ecological role and have a significant impact in medicine by the development of healthcare-associated infections. The National Institutes of Health (NIH) estimated that bacterial biofilms are involved in 65% of microbial diseases and in more than 80% of chronic infections (Jamal et al., 2018). Sessile cells can colonize indwelling medical devices as any type of catheters, contact lenses, heart valves, and protheses. Their presence on retrieved infected implants is easily detectable by laboratory methods. Indeed, bacterial colony outgrowths can be revealed by culturing techniques but can also be directly visualized by microscopy methodologies (Dibartola et al., 2017). Biofilm formation is equally involved in non-device-associated infections as periodontitis, osteomyelitis, and chronic infections (Srivastava and Bhargava, 2016). *Pseudomonas aeruginosa* biofilms are particularly deadly in cystic fibrosis (CF) patients. They also have a relevant impact on clinical outcomes of patients with chronic wounds (Mulcahy et al., 2014). Relevant animal models are now available to study the involvement of *P. aeruginosa* sessile cells *in vivo* infections. Diabetic wounds were mimicked in mice by Watters et al. (2013) and a porcine model allowed replicating the development of bacterial infections in CF lungs (Pezzulo et al., 2012).

A specific feature of sessile cells is their inherent tolerance to antimicrobials. Despite this basic knowledge, classical antibiotic susceptibility testing, providing the minimal inhibitory concentration (MIC) of molecules, is performed on non-adherent bacteria. Results collected according to antibiogram methods cannot predict the therapeutic success of the corresponding antibiotic therapies against biofilms. Furthermore, it is now well-recognized that low doses of antibiotics, encountered during continuous and fluctuating treatments, can stimulate biofilm establishment and are partly responsible for biofilm-specific antimicrobial tolerance.

Currently, no guidelines exist to help clinicians treat this kind of infections, although they are involved in the majority of untreatable clinical cases. Therefore, it appears urgent to develop a susceptibility test specific to biofilm or to validate a new-existing method for a routine use in diagnostic labs.

This review summarizes the basic knowledge about the growth of bacteria within a biofilm and the main steps of its formation. The tolerance features of sessile microorganisms to antimicrobial molecules were also detailed as well as the beneficial or deleterious effects of antibiotics for biofilm treatment. Available diagnostic tools for the selection of appropriate therapies against adherent bacteria are discussed herein.

## The Bacterial Biofilm

### A Community Way of Life

The growth of bacteria within biofilms is a natural process. The entirety of microorganisms could be sessile and live attached to a surface. This community mode is different from the planktonic growth, in which bacteria are isolated and mobile in the environment. The sessile cells differ from the planktonic ones by their morphology, physiology, and gene expression. The ability to adhere and grow on a surface as a biofilm is a survival strategy allowing the colonization of the environment by microorganisms. Bacteria continuously switch from a planktonic phenotype to a sessile one. This state variation is strategic for the cell as it allows a rapid adaptation to environmental conditions (Lebeaux and Ghigo, 2012).

The use of microscope can highlight a specific mushroom-like structure, especially for *P. aeruginosa* biofilms. They are mainly composed of microorganism clusters, delimited by aqueous channels. These latter separate bacterial microcolonies and allow the flow of oxygen and nutriments in the deepest areas of the biofilm as well as the elimination of degradation products. Nevertheless, it appears hard to generalize the composition, structure and features of biofilms owing to the wide range of environments and bacterial species. External factors, as medium composition and/or genetic properties of bacteria, contribute to the perpetual structure variation of the sessile population.

The key step of biofilm development is the synthesis of the extracellular matrix. It incorporates all the elements apart the bacterial cells. By forming up to 90% of its total organic matter, the matrix is the main structural component of the bacterial biofilm. It is highly hydrated and mainly composed of exopolysaccharides, proteins, nucleic acids, and minerals (Limoli et al., 2015). Its composition depends on the bacterial species and growth conditions. It allows strengthening of the biofilm structure while keeping a high flexibility. It also plays a protective role as it enhances the tolerance of bacteria to antimicrobials by creating a physical barrier that limits their diffusion to other environmental factors (UV, pH, and osmotic pressure variations, desiccation, etc.).

During the early development of the bacterial structure, it has been highlighted that extracellular DNA (eDNA) is essential for the adhesion of microorganisms and for their intercellular cohesion (Whitchurch et al., 2002). Quantitatively, in the biofilm matrix of *P. aeruginosa*, eDNA is six times more abundant than proteins and eighteen times more abundant than carbohydrates. Its origin was confirmed as being genomic. Nucleic acids can arise either from the lysis of a part of sessile cells or from an active secretion by living bacteria through merging membrane vesicles (Okshevsky and Meyer, 2015).

### Development of a Mixed Environment

Bacterial biofilm can be formed in a few hours. Its general development consists of five main steps (Dufour et al., 2010). Mobile free-floating bacteria detect an available conditioned surface through environmental signals as pH variation, oxygen and nutriment concentrations, temperature, and osmolarity, etc. They are transported by physical forces or bacterial appendages (i.e., flagella). The flagellum is as much required in the surface arrival as in the biofilm formation initiation since mutants defective in its synthesis are not able to adhere (O’Toole and Kolter, 1998). In the same way, a complete but inactive flagellum does not allow the establishment of the biofilm (Vallet et al., 2001).

**FIGURE 1:**
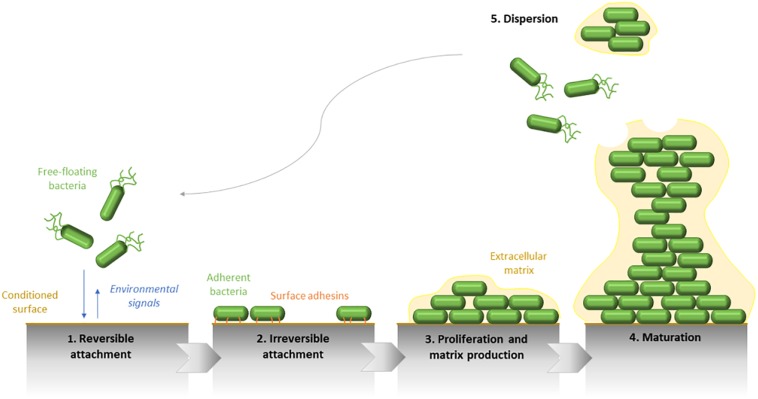
Schematic representation of the five main steps defining the *Pseudomonas aeruginosa* biofilm development. The biofilm formation begins by the initial attachment of mobile bacterial cells to the surface and is followed by the irreversible adhesion of bacteria, which form a monolayer along the surface. Therefore, biofilm maturation is characterized by the matrix production and the formation of three-dimensional structures. Finally, the biofilm dispersion reflects its life end.

The increasing proximity of the support, which is conditioned by the fluids and flows to where it is exposed, allows the initial adhesion of bacterial cells by physicochemical and electrostatic interactions. At this stage, the adhesion is reversible (Figure 1). Besides the environmental influence, this attachment is strongly influenced by the nature of the surface itself. A rough and/or hydrophobic surface boosts the adhesion of microorganisms, contrary to a smooth and/or hydrophilic support. Furthermore, the organic molecules, which are present at the surface, also condition the cell attachment.

Following this first step, which can occur few seconds after the initial contact with the surface, a second stage of adhesion happens, allowing the strengthening of the bacteria-surface bonds by the implication of bacterial compounds, such as type IV pili or more generally, surface adhesins. The surface binding, becoming irreversible, enables subsequently the multiplication of adherent bacteria, and the formation of microcolonies.

As for the flagella, *P. aeruginosa* mutants defective in the production of type IV pili adhere to a surface by the formation of a cell monolayer but are not able to gather in microcolonies. This data confirms that the microcolony formation is a process requiring bacterial mobility and not only a clonal growth from a bacterial cell (O’Toole and Kolter, 1998). In general, bacterial structures involved in the mobility of microorganisms are needed for the initial step of biofilm formation. They allow the approach and the colonization of the surface. The use of DNA microarrays on biofilms formed by the PAO1 strain, on sterilized granite pebbles in a continuous-culture model, showed that genes required for the synthesis of bacterial surface structures are repressed as soon as biofilm formation is initiated (Whiteley et al., 2001). They are no longer necessary for biofilm development and move on to compounds allowing its structuring and differentiation. It was shown that QS is involved in the structural steps of the biofilm. Analysis of *P. aeruginosa* wild type and *lasI* mutant highlighted an architectural difference in both biofilms (thinner and less heterogeneous for the mutant one) (Davies et al., 1998).

The first maturation step of biofilm development is defined by the production of the extracellular matrix. It allows a mechanical cohesion between bacterial cells and favors the switch from a “free life” to a “static life.” Its composition fluctuates in space and time and determines the spatial configuration of the biofilm.

As for the gene inactivation of extracellular structures, studies on *P. aeruginosa* showed that bacteria have a “sense of touch,” namely the ability to detect the presence of a surface and to combine a specific gene expression. During the first stages of adhesion, the transcription of genes involved in the alginate synthesis is activated to organize the matrix production after the formation of microcolonies (Davies and Geesey, 1995). More recent works showed that this gene regulation is directly dependent on the cyclic di-GMP (c-di-GMP), a central messenger present in the cytoplasm of bacterial cells, which controls the transition between the planktonic life and biofilm establishment and whose intracellular concentration is affected by environmental stimuli (Donné and Dewilde, 2015).

If growth conditions are optimal, a second phase of biofilm maturation occurs, defined by a growth in thickness. Therefore, the mature biofilm shows a complex 3D-structure. It can acquire a typical “mushroom-like” shape formed by bacterial columns on the basement of cells and in which bacteria are mobile. The whole biofilm is surrounded by an extracellular matrix. Through this structure, channels remain and allow the transfer of oxygen and nutriments required for the growth of sessile bacteria. Gradients of oxygen and pH also set up from the top to the bottom of the biofilm. These variations of concentrations within the biofilm lead to metabolic activity and growth differences of bacteria even in a monomicrobial community, their activity being increased at the surface and reduced at its center. These physicochemical differences lead to a physiological heterogeneity of microorganisms and generate the formation of environmental microniches constituted by bacterial subpopulations that are genetically identical but physiologically distinct (concerning the tolerance against antimicrobials for instance).

The final step of the biofilm formation cycle is its destructuring. Biofilm dispersion can be initiated by various factors as mechanical disruptions (abrasion), enzymatic degradations (enzyme secretion determined by QS) or even a lack in nutriments or an overpopulation (McDougald et al., 2011). Fractions of bacteria are removed from the community and are spread in the environment. Newly mobile and adherent individual cells will be able to explore and colonize new surfaces by the establishment of a new biofilm. A new cycle of adhesion/maturation can get back.

Biofilms must be considered as an elaborate and dynamic organization which constantly evolve to get used to its environment. The passing through the sessile state to the planktonic one plays a considerable role in the transmission of bacteria from environmental reserves to the host and also in the transmission between hosts and in the infection propagation in the individual (biofilm metastasis).

## Bacteria Under Shelter

The main advantage of the sessile way of life is the modification of the adherent bacteria in regard to their susceptibility to mechanisms of immune defenses and antimicrobials. Indeed, a single planktonic cell is vulnerable to the action of antibodies or phagocytes and is fairly sensitive to antibiotics. Conversely, bacteria that are embedded in a biofilm structure can be tolerant to the host immune system, antimicrobials, and biocide molecules (Høiby et al., 2011). Indeed, we usually talk about biofilm tolerance to antibiotics rather than resistance.

The resistance can be defined as the ability of a microorganism to grow when an antimicrobial compound is present in the environment. Resistance mechanisms are heritable and avoid the antibiotic interaction with its target. On the contrary, the term tolerance must be used for bacteria, which are able to survive in high concentrations of antimicrobials, but with a suspended growth. This feature, specific to the sessile bacterial life, is reversible, phenotypical, and non-inherited. A bacterial cell from a biofilm, which is resuspended in liquid medium, will get back an *in vitro* susceptibility to antimicrobials.

### Inefficiency of Immune System

The size of the bacterial biofilm is the first brake to the phagocytosis process. Even in immunocompetent individuals, components of the immune system are seldom effective against biofilm infections. During the innate immune response, macrophages and neutrophils are rapidly activated following the direct contact with bacteria (i.e., through the O-antigen of the bacterial lipopolysaccharide (LPS) or the alginate for *P. aeruginosa*). Then, the immediate immune response triggers an important accumulation of neutrophils around the biofilm structure associated with an oxygen exhaustion, which is due to an active stimulation of the oxidative metabolism, as the molecular oxygen is reduced in superoxide (Watters et al., 2016).

The phagocytic cells penetrate with difficulty the extracellular matrix. They are slowed down and become more vulnerable to the inactivation by bacterial enzymes. Besides, the extended lysis of neutrophils leads to the overflow of harmful compounds in the medium, which are responsible for consecutive tissue damages. The host immune response is the main cause of the healthy tissue degradation surrounding the bacterial infection.

Concerning the memory of the immune system response, it has been reported that specific antibodies against bacterial compounds as the elastase, the LPS or the flagellum are secreted in CF patients. These data show that antigenic determinants were neutralized during chronic lung infection. Unfortunately, it has been demonstrated that these antibodies contribute to the formation of immune complexes, which are precipitated into the parenchyma and lead to complement activation and opsonization of neutrophils, namely in an indirect way, to the nearby tissue degradation (Jensen et al., 2010).

### General Tolerance to Antimicrobials

Biofilm tolerance to external aggressions, notably to antibiotic treatments, is one of its exceptional features. It is well-known that the MIC of an antimicrobial, which is effective on sessile bacteria, is 10 to 1,000 times more concentrated that the one which would be active on their planktonic version (Schurek et al., 2012). This decreased antimicrobial susceptibility can have several causes. It can be inherent to the own organization of the biofilm (structure and functioning) but can also be acquired by transmission of resistance factors.

Given its complex architecture, the biofilm itself creates a protective environment for bacterial cells. It can be seen as an “innate” tolerance. As for compounds of the host immune system, the extracellular matrix forms a mechanical barrier limiting antibiotic diffusion within the biofilm and their access to microorganisms. The electrostatic charges or some matrix components bind antimicrobial molecules and trap them. The general high viscosity of the polymeric matrix can also prevent the antibiotics from reaching their effective concentrations in the deeper layers of the bacterial community. Consequently, bacteria in the outer layers of the biofilm die following an antimicrobial treatment, while those in the deeper layers have time to react (Paraje, 2011). This invasion delay should be enough to allow a progressive physiological adaptation of bacteria exposed to antimicrobials (expression of resistance genes, secretion of inactivating enzymes…). For instance, it has been demonstrated that alginate and eDNA in the biofilm matrix of *P. aeruginosa* could link aminoglycosides and play a role in the sessile bacterial tolerance to tobramycin (Hentzer et al., 2001). Similarly, an extracytoplasmic process of antibiotic sequestration by periplasmic glucans was highlighted. The locus *ndvB* was identified as being required for the production of cyclic glucans (Mah et al., 2003). The authors showed that polymers physically bound antimicrobial compounds in the periplasm, leading to the diffusion slowdown of antibiotics into the cells and preventing them from reaching their action sites. Nevertheless, this global diffusion barrier, specific of the biofilm matrix and the sessile cells, appears to be strain- and antibiotic-dependent. By itself, it cannot explain the radical tolerance of biofilms against antimicrobial agents.

In view of their own biofilm organization, bacterial metabolism plays an important part in antibiotic tolerance. The concentration gradients of metabolites, oxygen, and nutriments within the mature biofilm create bacterial niches that are less metabolically active. For example, in *P. aeruginosa* microcolonies, the oxygen is consumed faster at the surface than it diffuses into the deeper layers of the biofilm. Its graduated diffusion leads to the formation of hypoxic areas in the bacterial community (Serra and Hengge, 2014). Some of microorganisms could get back to a stationary phase in lowering their growth and multiplication rates as an induced stress response. This reduced metabolism of sessile cells is partly responsible for the tolerance associated with the biofilm, as the action mode of the majority of antimicrobials targets metabolic processes in growing bacteria (replication, transcription, translation, or cell wall synthesis). Lots of works have validated the advantageous efficacy of antimicrobials on active bacteria, which are located in external areas of the biofilm. However, parallel studies showed that other types of molecules, such as SDS, EDTA, or chlorhexidine could conversely act on bacterial cells in stationary phase of growth, located in the internal niches (Ciofu et al., 2015).

The activation of efflux pumps by bacteria embedded in the extracellular matrix can also contribute to the inefficiency of antimicrobials in actively discharging them outside the biofilm structure before they can reach their target. These membrane transporters can be specific of a class of antibiotics or responsible for multidrug resistance. In Gram-negative bacteria as *P. aeruginosa*, efflux pumps are usually composed of a pump located in the inner membrane, an outer membrane factor, and a periplasmic fusion protein. The association of cell impermeability with the expression of the efflux system MexAB-OprM leads partly to the inherent resistance of the bacillus to antibiotics. The expression of some of them was demonstrated as being specific to the biofilm mode (Zhang and Mah, 2008).

The bacterial density and the spatial proximity of microorganisms within a mature biofilm promote the gene exchange and the resistance plasmid transmission. The horizontal gene transfer could be 1,000-fold more important in a bacterial community than between planktonic cells. Due to the starved local environment within the biofilm, bacteria are also subjected to random mutations and genetic rearrangements. This generation of bacterial variants, favored by natural selection, leads to a chromosomal resistance (Poole, 2012). The mutation frequency can be stimulated by environmental factors, as the presence of reactive oxygen species from the lung inflammatory response. These reagents, in damaging DNA, cause mutations in bacteria and lead to the diversity of bacterial phenotypes in the biofilm (Rodríguez-Rojas et al., 2012). Finally, by combination of several of these mechanisms, sessile bacteria rapidly became multiresistant.

It also demonstrated the existence of a “persister” bacterial population which could constitute a reserve allowing the infection relaunch after elimination of peripheral planktonic and sessile cells (Stewart, 2002).

### The “Persister Cells” Enigma

Persisters are regular cells exhibiting a specific non-growing phenotype, combined with an excessive tolerance to antibiotic concentrations. Their existence was firstly described in the 1940s (Bigger, 1944). The transcription downregulation of genes involved in motility and energy production was highlighted for these isolated bacteria. Consequently, as they are in a dormant state, antimicrobials are able to bind to their target molecules but not to impair their initial function (Lewis, 2005).

The presence of persisters can be easily detected in bacterial cultures by a process of biphasic death, further to an exposition to bactericidal antibiotic concentrations. Firstly, a lethal dose of antimicrobials will rapidly eradicate the sensitive bacterial population. A much slower second phase of death follows, reflecting the poor killing of persister cells. Finally, the end of antimicrobial treatment will allow the renewal of the bacterial community by regeneration of persister survivors (Conlon et al., 2015).

Each bacterium shows the capacity to be differentiated in a persister cell, but few of them are really observed during the early exponential phase of growth. Indeed, the genuine persister population is formed during the mid-exponential phase and finally, they reach up to 1% of the overall population at the stationary phase. This phenotypic conversion can be induced by environmental stimuli or stresses, as antibiotic exposure, which are predictive of immediate threats for cells, or they may be preexisting in the bacterial population (Harms et al., 2016; Fisher et al., 2017). It is assumed that stochastic modifications in genes can lead to the phenotypic switch along with the over-expression of specific toxin-antitoxin (TA) module proteins. Typically, the toxin portions are neutralized by their antitoxins, but under cellular stresses, proteases must be over-expressed and degrade the antitoxin proteins. In that case, the toxin modules are free to exert their toxic action on bacteria. The expression of many other compounds implicated in the persister phenotype can be induced by environmental stimuli. The signaling pathway of the SOS response and the alarmone ppGpp, two stringent responses to stress, also appears to be associated with the persistence of bacteria (Del Pozo, 2018).

## Effectiveness of Conventional Antibiotics

Despite the intensive tolerance of the biofilm to antimicrobials, certain conventional antibiotics still demonstrate activity against bacterial cells growing in the biofilm state.

In a recent study, Otani et al. (2018) showed that sub-MICs of ceftazidime reduce biofilm volume, inhibit twitching motility, and repress gene expression involved in bacterial adhesion and matrix production of *P. aeruginosa* PAO1. Roudashti et al. (2017) had previously noticed this effect of cephalosporin on motility and biofilm formation for the same strain.

Similarly, other common antimicrobials were described as being effective on biofilm behavior of *P. aeruginosa*. Subinhibitory doses of piperacillin/tazobactam altered the pathogenic potential of various clinical and laboratory strains of *P. aeruginosa* in reducing bacterial adhesion, in decreasing biofilm formation, swimming, and twitching motility and conversely in increasing the susceptibility of cells to oxidative stress (Fonseca, 2004). Indeed, one early step of biofilm formation which can be targeted for the prevention of chronic infection is bacterial adhesion to a surface. The process of twitching motility contributes to this part of virulence of sessile microorganisms. Wozniak and Keyser (2004) noticed that clarithromycin substantially inhibited cell translocation of *P. aeruginosa* through its type IV pilus as well as altered its biofilm architecture at sub-MIC levels. Another control strategy against bacterial biofilm is QS disruption. Azithromycin, ceftazidime, and ciprofloxacin showed, at subinhibitory concentrations, QS-inhibitory activities in bacteria (Skindersoe et al., 2008). This beneficial effect of the macrolide was also emphasized in an experimental urinary tract infection model. Antibiotic concentrations below the MIC could inhibit the production of QS molecules, leading to the complete clearance of *P. aeruginosa* from the mouse kidneys (Bala et al., 2011). Azithromycin was also described as being able to prevent PAO1 biofilm formation in a flow cell biofilm model (Gillis and Iglewski, 2004).

Continuous treatment of colistin (25 μg/ml) turned out to be effective against the non-dividing central part of a *P. aeruginosa* biofilm growing in a flow chamber for 4 days. Associated with ciprofloxacin (60 μg/ml), which can kill metabolically active cells in the surface layers, this combination therapy showed a clinical efficacy for the early eradication treatment of bacteria in CF patients (Høiby et al., 2010). In a more recent study, the association of minimal biofilm inhibitory concentrations (MBICs) of fosfomycin with tobramycin (≥1024 μg/ml and from 8 to 32 μg/ml, respectively) has been demonstrated to be synergistic against CF isolates in *in vitro* models (Díez-Aguilar et al., 2018). Overall, the aminoglycosides usually prescribed in CF (amikacin and tobramycin) showed a preventive action on the early adhesion of clinical *P. aerugin*osa strains at various concentrations (sub-MICs, MICs, and so-called PK/PD doses) (Olivares et al., 2017).

The efficacy of antibiotic lock solution (meropenem, levofloxacin, and colistin) on *P. aeruginosa* clinical and reference strains was also confirmed. The antibiotic lock technique (ALT), using antimicrobial molecules, prevented bacterial regrowth in an *in vitro* antibiotic lock model. The efficacy of ALT to eliminate *P. aeruginosa* biofilms should be improved when the three antibiotics were used in combination with clarithromycin (Ozbek and Mataraci-Kara, 2016).

Finally, all the cited publications attest that some conventional molecules can still be active on *P. aeruginosa* in the context of chronic infections, in preventing its growth within the biofilm. Nevertheless, a lot of these studies were carried out on the reference PAO1 strain. To confirm the clinical effectiveness of antimicrobial treatments, antibiotic susceptibility testing must be performed on clinical isolates, and clinical trials must be planned.

Concerning the positive effect of classic antimicrobial therapies on other sessile pathogens, MICs and minimal bactericidal concentrations (MBCs) of rifampicin have demonstrated an activity against biofilms of *Staphylococcus epidermidis* and *Staphylococcus aureus* isolates associated with device infections, especially when it is used in association with other molecules as fusidic acid, vancomycin or ciprofloxacin in an *in vitro* biofilm model (Saginur et al., 2006). More recently, adherent staphylococci involved in skin and soft tissue infections were described as being more or less susceptible to multiples of tedizolid MICs and other comparator agents (vancomycin, linezolid, and daptomycin) (Delpech et al., 2018). The use of daptomycin-lock therapy (50 mg/ml) also showed a therapeutic advantage for the 24 h-treatment of a long-term catheter-related bloodstream infections by coagulase-negative *S. epidermidis* in a rabbit model (Basas et al., 2018). Similarly, subinhibitory concentrations of fluoroquinolones were able to reduce the number of sessile cells to prevent the adhesion of the corresponding *S. epidermidis* strains and to alter biofilm morphology (Szczuka et al., 2017).

This overall review of publications, dealing with the anti-biofilm property of conventional antimicrobials, can be completed with studies using *Escherichia coli*, another bacterial specie well-characterized for its capacity to form biofilm structures. In a recent article, Klinger-Strobel et al. (2017) noticed that colistin concentrations from 4 to 16 mg/l could reduce the amount of adherent *E. coli* bacteria and exert a matrix-reducing effect on biofilms in formation. Similarly, Butini et al. (2018) investigated the anti-biofilm property of gentamicin-eluting bone graft substitute against bacterial species involved in bone and implant-associated infections. Calcium sulfate bone graft substitutes served as local antibiotic delivery carrier, and gentamicin is one of the most used molecules for the treatment of bone-related infections. Therefore, they demonstrated that 12 μg/ml of released gentamicin were able to prevent *E. coli* adhesion and 23 μg/ml of the molecule could eliminate a 24 h-old biofilm. These data are promising as the applied concentrations are achievable for local treatment in bone and soft tissues. In another recent publication, a mupirocin spray was formulated and tested against *E. coli* strains, in a context of wound and surgical site infections. Inhibition and disruption of formed biofilms were achieved with a single and a sub-actual dose of the antibiotic spray (1 mg per spray or 1 mg per 50 mg of ointment), compared to the commercialized mupirocin ointment (Bakkiyaraj et al., 2017). Results showed that both formulations had an anti-biofilm action on *E. coli* sessile cultures in tissue culture plates but microscope studies provided complementary evidences that it remained more individual adherent cells with ointment formulation than for treatment with the antibiotic spray. Authors concluded that this efficacy on biofilm prevention and disruption was comparable with that of the ointment. Nevertheless, the spray use seemed beneficial as it included an easy application: while the ointment was removed from the application site upon washing, the spray formulation significantly resisted removal after a single wash.

Finally, for the treatment of recurrent urinary tract infections (UTIs) associated with the presence of biofilm, the use of amikacin, ciprofloxacin, and third-generation cephalosporins could be recommended. Indeed, various concentrations of these molecules, selected according the bioavailability of antibiotics in human urine, showed the ability to significantly reduce biofilm biomass in a study of 116 *E. coli* strains of UTIs (González et al., 2017).

Even if it seems that antimicrobials can be effective on prematurely adherent bacteria, a small percentage of persister cells develop a high tolerance to antibiotics and are typically involved in infection relapses. Moreover, the biofilm must be considered as a single compartment with its own pharmacokinetic parameters. This will influence local antibiotic concentration and its metabolization in the biofilm (Cao et al., 2015). All these combined factors lead to the recommended use of high doses of antimicrobials for long periods, which cannot always be practicable in patients because of severe systemic side effects.

Biofilm tolerance to antimicrobials is complex and above all multifactorial. A now long list of studies demonstrate that low doses of antimicrobials at the infection site might increase the selection of mutagenesis and the risk of biofilm formation initiation induction (Ciofu et al., 2017).

## Induction of Biofilm Formation by Antimicrobials

At high concentrations, antibiotics appear to be perfect bacterial killers. Their original function in the environment is fighting and inhibiting the growth of competitors (Linares et al., 2006). The production of antimicrobials by bacteria themselves allows the killing of predators. This application of the classical Darwinian principle supports the idea that they allow the competing colonization of soil by microorganisms. However, antibiotic molecules constitute only a small part of organic compounds produced by bacteria. Consequently, it can be assumed that they can affect the general modulation metabolic function in bacteria, as other signaling analogous (rhamnolipids, peptides, and QS signals, etc.). Supporting this assumption, phylogenetic analyses revealed that antimicrobial resistant genes were present in bacterial genomes millions of years before the modern use of antibiotics. A similar example concerns the metagenomic study of Alaskan soils, which demonstrated the existence of an ancient and varied collection of β-lactamase genes, whereas this antimicrobial family is not detected in the environment (Aminov, 2009).

In activating specific gene transcription, antimicrobial compounds seem to act as signaling molecules, regulating the homeostasis of bacterial communities. As sessile cells are significantly less sensitive to antimicrobials, biofilm formation would be a strategic evolution of bacterial populations to counteract non-lethal doses of antibiotics produced by soil microorganisms. This implies that antimicrobials can also be beneficial for the survival of susceptible planktonic cells in nature. Therefore, they can permit a more efficient colonization of heterogeneous environments. Especially at subinhibitory levels, antibiotics modulate bacterial virulence, stress response, motility, and biofilm formation (Song et al., 2016).

The first report describing the ability of low doses of antibiotics to interfere with bacterial functions was made by Gardner (1940). In the presence of subinhibitory concentrations of penicillin, numerous gram-negative, and positive pathogens formed elongated filaments.

Since then, similar studies introducing the structure modification of bacterial cells by antibiotics were published. *P. aeruginosa* showed the most morphological changes as bacteria reacted to meropenem and biapenem in forming a “bulge” midway along them (Horii et al., 1998). The authors also described a relationship between the induction of a new morphology and the amount of endotoxin released by bacteria. The modification of the bacterial size and shape was explained by the fact that antibiotic molecules inhibit the Penicillin-Binding-Proteins 2 and 3 (PBP-2 and PBP-3). They are involved in the assembly of the bacteria cell wall by the catalysis of the terminal stages of the peptidoglycan network. These proteins are the primary target of β-lactam antibiotics in Gram-negative bacteria. The inactivation of PBP-1 leads to bacteria lysis, whereas the inhibition of PBP-2 and PBP-3 is associated with either spherical cells or filamentous bacteria. These effects of antimicrobials onto the bacterial morphology were also observed in experimental infections in mice (Yokochi et al., 2000). More precisely, the authors highlighted a relationship between the shape of bacteria and their susceptibility to phagocytosis. Induced round bacteria were phagocytosed by peritoneal cells, whereas long filaments were not. Finally, it appeared that the morphological reorganization of bacteria is a reversible process as when antibiotics were no longer present, the induced spherical or filamentous population converts back to the normal bacillary form (Monahan et al., 2014). This transition is a strategy to survive antibiotic exposure as the biofilm formation.

Furthermore, exposure of *P. aeruginosa* strains to sub-MICs of ciprofloxacin leads to the selection of pre-existing mutants with high level resistance (Jørgensen et al., 2013). The analysis of the strains selected by subinhibitory doses of the fluoroquinolone showed phenotypic changes in bacteria, as decreased protease activity and swimming motility, down-regulation of the type III secretion system and higher levels of quorum-sensing signals (Wassermann et al., 2016). In some cases, this implies that antibiotic therapies may expose patients to detrimental side-effects by accelerating pathogen adaptation and raising the risk of antimicrobial tolerance and spread in the commensal bacterial flora if bacteria are exposed to low antibiotic concentrations.

The first study demonstrating the real “inducer” property of antimicrobials on biofilm formation at subinhibitory concentrations dates back to 1988 (Schadow et al., 1988). The adherence of coagulase-negative staphylococci was increased to 65% after rifampicin treatment. Since then, numerous works focusing on the effect of low antibiotic doses on biofilm formation were published.

Although several studies have shown that aminoglycoside antibiotics act as antagonists of biofilm formation *in vitro* (see previous section of this paper), opposite data were collected and highlighted the ability of the same molecules to significantly induce the sessile growth of a variety of bacterial species. Hoffman et al. (2005) described the effect of subinhibitory doses of aminoglycosides in *P. aeruginosa* and *E. coli* biofilms. They showed that these antibiotics stimulated the expression of the *arr* gene, which encodes a phosphodiesterase whose substrate is the c-di-GMP. Reinforcing this idea, induction of biofilm formation after an exposure to sub-MICs of aminoglycosides was detected among half of a *P. aeruginosa* clinical isolate collection (Elliott et al., 2010).

Imipenem, a carbapenem molecule, was also able to substantially influence the expression of 34 genes in the common reference PAO1 strain. When subinhibitory concentrations of this antibiotic were applied to bacterial cultures, the alginate gene cluster, the main component of the biofilm matrix of *P. aeruginos*a was more than 10-fold induced (Bagge et al., 2004). The corresponding polysaccharide amount in biofilms was quantified by the authors and they found that the alginate level in the matrix of stimulated-biofilms was 20-fold higher than the one of the non-exposed controls.

Induction phenomenon of biofilm formation by antimicrobials was also described for other bacterial species. The use of β-lactam antibiotics at sub-MIC concentrations leads to the induction of the bacterial adhesion by a community-associated Methicillin-Resistant *S. aureus* (MRSA) strain and clinical isolates (Ng et al., 2014). All of the antimicrobial molecules tested in this study exhibited a biphasic dose-response curve. Authors also described an inversely proportional relationship between the biofilm amount and the susceptibility of bacteria to methicillin: the more sensitive the strain to antibiotic, the lower is the concentration required to induced biofilm. Biofilm formation of a clinical isolate of *Enterococcus faecalis*, which commonly underlies prosthetic valve endocarditis and multiple device infections, was also significantly increased by low concentrations of ampicillin, ceftriaxone, oxacillin, and fosfomycin. This enhancement of biofilm establishment appeared to be specific of molecules inhibiting cell wall synthesis (Yu et al., 2017). Additionally, a collection of ninety-six clinical isolates of *S. epidermidis*, which originate from various samples as wounds, catheters, sputum, etc., was recovered by He et al. (2016). The authors described that 27% of erythromycin-resistant strains exhibited biofilm induction by 0.25 MIC of the molecule. The induction intensity ranged from 1.11-fold to more than twofold (He et al., 2016).

A more complete review article written by Kaplan (2011) gathers studies showing that subinhibitory concentrations of antimicrobial molecules can act as agonists of bacterial biofilms *in vitro*.

Biological responses of bacterial species are strain- and dose-dependent. Some molecules can promote the biofilm formation at high levels and conversely be antagonists and repress its establishment at lower doses. Nonetheless, this dose-response relationship cannot be generalized as it can be inverted, depending on the considered antibiotic, which is used in the treatment for a specific laboratory or clinical strain. The discovery of this ecological function of antibiotics is essential as, in a clinical context, the induction of biofilm formation by low concentrations of antibiotics would contribute to the failure of antimicrobial therapies in case of biofilm-related infections. It can be speculated that this is a common phenomenon as microorganisms are usually under fluctuating doses of antibiotics during a chemotherapy.

### Clinical Tools Available for the Diagnosis and Treatment of Biofilm Infections

Traditionally, clinical microbiology laboratories have focused on the culture of isolated bacterial strains and provide their susceptibility to antibiotics in defining the breakpoints and the PK/PD parameters under planktonic growth conditions. The corresponding antibiotic therapies, based on non-adherent microorganisms, are often associated with treatment failures and/or recurrence of the infection. No guidelines are offered to clinicians to successfully treat biofilm infections, which can result to false-negative data if the samples do not significantly represent the main infection.

Besides, there is still no available standardized tool to detect easily the presence of sessile cells in a clinical sample and allow determination of their specific antibiotic susceptibility. As biofilm bacteria are inherently more tolerant to antimicrobials, the establishment of the corresponding breakpoints to predict therapeutic success is needed. New methods monitoring the effect/response of biofilm cells to antibiotic therapy must be designed. Currently, two technologies were developed but not yet standardized for a rapid routine use in hospital laboratories: the Calgary device (also called MBEC assay) and the Antibiofilmogram^®^.

**FIGURE 2:**
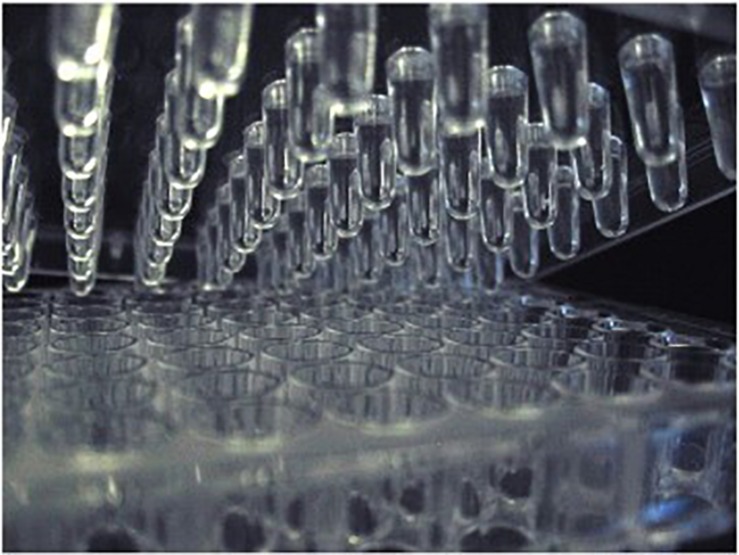
Regular MBEC with 96-well plate. Photography adapted from Parker et al. (2014).

The Calgary biofilm device consists of a two-part reaction vessel (Ceri et al., 1999). A lid, composed of 96 pegs, forms the first and top component. The bottom component of the device is a standard 96-well plate, in which the pegs are designed to sit into each well. The microplate contains the medium allowing the growth of bacteria, which is set up in 96 equivalent biofilms at each peg site (Figure 2). Biofilm susceptibility testing is performed in transferring the lid with bacterial biofilms to a standard 96-well plate containing serial dilutions of antibiotics. The minimal biofilm eradication concentration (MBEC) is defined as the minimal concentration of antibiotic that prevents visible growth in the recovery medium used to collect sessile cells.

Moskowitz et al. (2004) evaluated the *in vitro* activity of twelve antimicrobials on a large number of CF sessile clinical isolates of *P. aeruginosa* using a modified Calgary device protocol. The MBICs of the antimicrobial agents were much higher than the corresponding conventional MICs. In a context of biofilm infections, this suggests that the use of broth microdilution susceptibility testing or other standard methods to guide therapy may not contribute to improve clinical outcomes. Quite the reverse, devices simulating biofilm growth conditions might guide therapy more effectively. To verify this hypothesis, clinical trials were already conducted. Unfortunately, no evidence that biofilm susceptibility testing performed with the Calgary device was more efficient than conventional techniques in terms of clinical outcomes was provided (Waters and Ratjen, 2017). Yau et al. (2015) introduced the first randomized controlled trial evaluating the utility of biofilm antimicrobial susceptibility testing in the treatment of CF pulmonary exacerbations. They concluded that the choice of antibiotic therapies based on biofilm behavior of bacteria did not improve clinical outcomes and did not decrease pulmonary bacterial loads. They explained the lack of Calgary method efficacy by an oversimplification of the sessile growth conditions. Biofilm formation on a lid composed of 96 plastic pegs could not recreate the environment *in vivo*, in which sessile cells grow and express specific properties. Moreover, the determination of antimicrobial susceptibility of a selected isolate could underestimate the microbial diversity response to antibiotics.

The Antibiofilmogram^®^ (ATBFG) method was specifically designed to investigate early steps of biofilm development, by rapidly screening antibiotic effective against sessile bacteria. Its functioning principle is based on the potential immobilization of magnetic microbeads by bacteria forming a biofilm in the well bottom of a microplate (Chavant et al., 2007).

**FIGURE 3:**
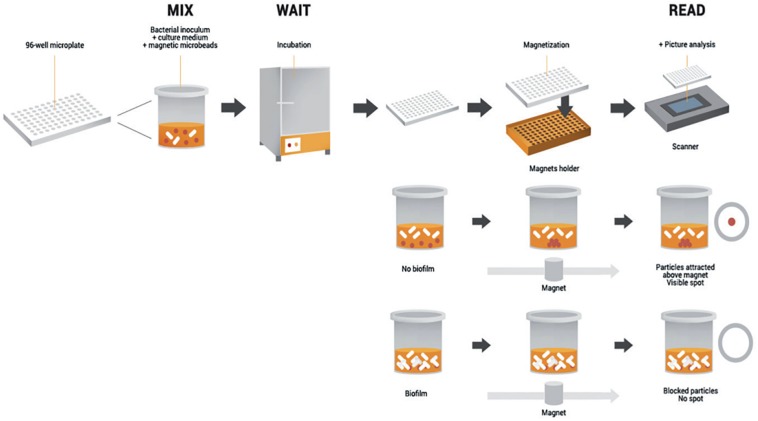
Schematic representation of the Antibiofilmogram^®^ principle. The initial bacterial suspension is loaded in a 96-well microplate with the microbead solution. After incubation, the plate is magnetized during 1 min. If bacterial cells preserve a free-floating form, the beads are attracted by the magnetic field and form a spot. Conversely, if bacteria adhere to the well bottom, beads are embedded in the biofilm in formation and consequently, no spot is visible. Schematic adapted from Azeredo et al. (2017).

Briefly, a given bacterial culture is mixed with the microbead suspension and loaded in the plate (Figure 3). Afterward, it is incubated and submitted to a magnetic field at a desired time point, without staining and/or washing stages. The formation of a brown spot in the bottom of wells reveals the free migration of beads during plate magnetization and so, the remaining-free state of bacteria. Conversely, the absence of visible spots reflects the bead blockage by a pre-forming biofilm (Olivares et al., 2016). The main advantage of this methodology is its capacity to collect data within a couple of hours, allowing comparison of antibiotic susceptibility of sessile bacteria to the results of classical antibiograms. But as for the Calgary method, the ATBFG is an *in vitro* assay, which does not provide information about structure or thickness of the mature biofilms.

Results of ATBFG performed on 29 clinical strains of *S. aureus* isolated from bone and joint infections (BIJs) were also published (Tasse et al., 2016). On the basis of antibiotic breakpoint values, the authors defined effective antimicrobial molecules against adhesion of the majority of *S. aureus* strains (rifampicin, linezolid, clindamycin, and fusidic acid), others inefficient against bacterial adherence (fosfomycin, ofloxacin, daptomycin, and gentamicin) and some of them whose efficacy was strain-dependent (cloxacillin, vancomycin, and teicoplanin). Data validity was confirmed by *in vivo* assays (catheter-related infections in the mouse). Results showed that serum concentrations of cloxacillin, corresponding to the MBICs determined by ATBFG (either 2 or 4 μg/ml), allowed reduction to 3 log the bacterial biomasses colonizing the catheters for three clinical strains, whereas the simple MICs of the antibiotic were inefficient on biofilm formation.

In a CF context, the use of ATBFG on clinical *P. aeruginosa* strains also showed the capacity of two aminoglycosides (amikacin and tobramycin) to prevent bacterial adhesion at concentrations close to the MICs (Olivares et al., 2017). Only *in vitro* assays were performed in this study but an inter-method reproducibility was conducted through Crystal Violet staining and a tissue culture system, which validated the inhibitory effect of the antimicrobials on the early adhesion of *P. aeruginosa* isolates.

## Concluding Remarks

As demonstrated in previous sections, the treatment of bacterial infections with chemically distinct antibiotics can lead to a variety of responses from sessile bacteria. Despite the increased tolerance of microorganisms toward antimicrobials, some molecules are always effective against newly adherent bacteria. In clinical practice, it is recommended that when possible, as for the diabetic foot for instance, to resort to topical administration to provide high local concentrations to the infection site without systemic side-effects.

Disappointingly, numerous studies have also described that low doses of antibiotics can significantly induce biofilm formation *in vitro* for a variety of bacterial species. The plausibility of this phenomenon *in vivo* must be considered as bacterial pathogens are exposed to sub-MIC concentrations of antimicrobials during a clinical therapy with fluctuating dosing regimens. More researches on antibiotic-induced biofilm formation are required to elucidate the involved mechanisms. Clinical trials that verify the relevance of this process in patients and the potential relationship with therapy failure will also be highly helpful. The prospect of complementary assays evaluating the susceptibility of free and sessile cells to antibiotics would also allow the optimization of the general use of antimicrobials in the treatment of biofilm-related infections.

## Author Contributions

EO wrote the manuscript. All authors read and approved the submitted version.

## Conflict of Interest

EO, CP, and TB were employed by the company BioFilm Pharma SAS, and SB-B, CP, and TB were employed by the company BioFilm Control SAS.

The remaining authors declare that the research was conducted in the absence of any commercial or financial relationships that could be construed as a potential conflict of interest.
